# Effectiveness of saline water and lidocaine injection treatment of intractable plantar keratoma: a randomised feasibility study

**DOI:** 10.1186/s13047-021-00467-7

**Published:** 2021-04-13

**Authors:** Marie-Philippe Mercier, Virginie Blanchette, Vincent Cantin, Magali Brousseau-Foley

**Affiliations:** 1 Department of Physical Activity Sciences and Podiatric Medicine, Université du Québec à Trois-Rivières, 3351, boul. des Forges, C.P. 500, Trois-Rivières, G9A 5H7 Canada; 2 Centre intégré universitaire de santé et de services sociaux de la Mauricie et du Centre-du-Québec (CIUSSS-MCQ) affiliated to Université de Montréal, Faculty of Medicine, Trois-Rivières Family Medicine University Clinic, 731, rue Ste-Julie, 2nd Floor, Trois-Rivières, G9A 1X9 Canada

**Keywords:** Debridement, Intractable plantar keratoma, Callosities; Injections, Saline solution; Lidocaine

## Abstract

**Background:**

An intractable plantar keratoma (IPK) is a conical thickening of the epidermis’ stratum corneum and a common cause of foot pain which can have a significant, detrimental impact on the mobility, quality of life and independence of individuals. Conservative treatments are currently offered to patients with IPK, but they are unsatisfactory since they do not offer a sufficient or permanent reduction of symptoms. The purpose of this study was the evaluation of the feasibility, safety and effectiveness of innovative treatments for intractable plantar keratoma (IPK).

**Methods:**

A randomized single blind trial with 40 patients divided with block randomization in four parallel groups was conducted to compare treatment combinations: conservative sharp debridement only or sharp debridement with needle insertion, physiological water injection or lidocaine injection. All patients obtained the same treatment four times at a four-week interval. At each visit, visual analog scale (VAS), Foot Function Index (FFI) and IPK size were evaluated. VAS and FFI were also completed at a six and twelve-month follow-up.

**Results:**

Our findings in regards to feasibility demonstrated recruitment challenges because of the anticipated pain that would be provoked by needle insertion may not be worth the potential pain relief compared to debridement alone from the patient’s perspective. This was also the principal cause of drop out. Our preliminary results show no main effect of group for any of the clinical outcomes: pain felt on VAS, FFI score, IPK’s size (*p* > 0.05). However, the analysis revealed a statistically significant effect of time on VAS (*p* < 0.001), FFI score (*p* < 0.001) and IPK’s size (width and depth (*p* < 0.001); length (*p* = 0.001)), but no group x time interaction was found (*p* > 0.05).

**Conclusions:**

This study demonstrates that IPK treatment consisting of sharp debridement with needle insertion, physiological saline water injection or lidocaine injection is feasible and safe. There was a non-statistically significant trend toward diminishing pain intensity compared to scalpel debridement alone. The pain provoked by needle insertion and injection treatments must be addressed with a scientifically proven protocol to make it more comfortable for patients before these treatments could be considered in further studies.

**Trial registration:**

ClinicalTrials.gov, NCT04777227. 2 March, 2021 - Retrospectively registered (All participants were recruited prior to registration).

**Supplementary Information:**

The online version contains supplementary material available at 10.1186/s13047-021-00467-7.

## Background

Callosities are a common cause of foot pain which can have a significant, detrimental impact on the mobility, quality of life and independence of individuals [1–5]. A common lesion in the callosities’ family is the intractable plantar keratoma (IPK). The IPK is a dermatologic painful lesion which consist in a conical thickening of the epidermis’ stratum corneum [6] on the plantar aspect of the foot [6]. This dermatological condition has a prevalence between 51 and 68% among people aged 65 and over and is a frequent consultation reason in medical clinics [7]. The elderly population is more susceptible to callosities because the loss of soft tissue is part of the aging process and atrophy of the plantar fat pad increase the plantar pressure, the pain and it limits ambulation [8–10]. The onset of this lesion can rarely be secondary to a genetic deficiency but is, in most cases, due to repetitive trauma caused by major pressure or friction points [6, 11, 12]. As an outcome of trauma, corneocytes which are terminally differentiated keratinocytes, have a higher speed of differentiation which leads to an incomplete differentiation [13]. This maintains the cells in contact with each other and preclude their desquamation from the epidermis, which causes callus accumulation and creates an IPK [14]. IPKs are mostly found in women, in people who spend long hours standing and in people having foot deformities that modify pressure points like bunions and hammer toes [3]. Despite the different types of treatment currently available for IPKs (keratolytic ointments, partial offloading with orthotic devices, insoles, paddings and therapeutic shoes, moisturizers and emollients, bleomycin sulfate or hyaluronic acid or silicone injections), the treatment of choice is scalpel debridement alone [4, 15–25]. Conservative treatment are currently offered to patients with IPK, but they are unsatisfactory since they do not offer a sufficient or permanent reduction of symptoms [26, 27]. If conservative treatments fail, surgical treatments such as arthroplasty, bunionectomy, osteotomy, skin flap and punch biopsy are offered [21, 27–29]. However, it has been reported that IPK’s surgical management can lead to transfer lesions because plantar pressure points are relocated [27, 28].

Evidence is scarce especially for IPKs management in the literature even if it is a widespread painful problem. A previous study at our institution compared the effect on pain relief and functional capacity of a subcutaneous injection of physiological saline water (PSW) compared to a subcutaneous injection of hyaluronic acid (HA) under a debrided IPK [20]. Considering this previous work, efforts are done to find innovative treatments for those who are suffering of an IPK. Therefore, the first objective of this study was to determine the feasibility and safety of four different treatments for painful IPK with scalpel debridement: alone, combined with needle insertion or a subcutaneous injection of PSW or a subcutaneous injection of lidocaine solution (LS). The second objective was to obtain preliminary result concerning the short and long term efficacity of these treatments on pain intensity scores. We hypothesize that treatments by needle insertion, PSW injection or LS injection of IPKs are feasible and safe and may show improvement of the IPK lesion on short and long term better than the debridement alone.

## Method

### Data source, recruitment and sample

**Fig. 1:**
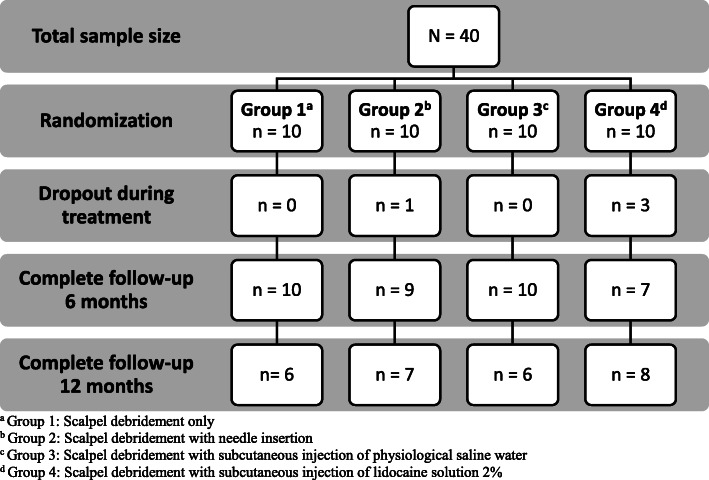
Patient flow diagram

This single blind feasibility study focused on safety and outcomes of an injection therapy for IPKs and was conducted in a private podiatric medicine clinic (Clinique Podiatres Plus) located in Quebec City and at the University of Québec at Trois-Rivières podiatry teaching clinic, in the province of Quebec, Canada. The study protocol was approved by the institutional ethical committee (UQTR CER-15-214-07.17). Initial protocol was amended in order to improve enrollment rate with use of publicity in local newspaper and add an additional recruitment clinical site (Clinique Podiatres Plus). Initial recruitment was realized by the professionals and the students in both settings as well as through a local newspaper advertisement. A total of 63 patients were approached to join the study. Forty patients met inclusion criteria which were verified by the principal investigator (MPM). Selection questionnaire is available in Additional file 1. All patients signed an informed consent form before their enrollment. The study was conducted on a 39-month period, between June 2015 to September 2019. Inclusion and exclusion criteria are listed in Table 1. Enrolled patients were informed that all treatments were free of cost, and if an intervention was significatively more effective, they would be offered this treatment at the end of the trial. A flow diagram summarizes patients’ distribution in Fig. 1. No minimal sample size calculation was done because of the nature of the study but a threshold between 40 to 60 patients was targeted according to the previous work on sample size for feasibility study that suggest a range of 10 to 300 participants with a median of 38 [30].

**Table 1:** Inclusion and exclusion criteria

**Inclusion criteria**	
• ≥ 18 years old	
• Having a painful IPK for at least 3 months	
**Exclusion criteria**	
• Ongoing pregnancy or breastfeeding	
• Severe cardiovascular or neurological disease	
• Immunosuppressed status	
• Presence of a plantar ulcer	
• Allergy to lidocaine	
• History of keloid or hypertrophic scar	
• Simultaneous painful plantar syndrome unrelated to the presence of an IPK	

Group 1: Scalpel debridement only.

Group 2: Scalpel debridement with needle insertion.

Group 3: Scalpel debridement with subcutaneous injection of physiological saline water.

Group 4: Scalpel debridement with subcutaneous injection of lidocaine solution 2%

### Randomization and blinding

Treatment allocation was made by block randomization for four parallel groups following a computer-generated list of random numbers (allocation ratio 1:1:1:1). Patients were assigned to a number in order of subscription. Patients were blinded to their treatment regimen during the whole study. Only independent research assistants had access to this list to prepare the treatment material in a covered container to avoid any bias during evaluation. The container was open once the podiatrist (MPM) had completed the physical evaluation and had confirmed a clinical IPK diagnosis [6]. All syringes were covered with medical tape (Hypafix, BSN) to mask their content.

### Intervention description

Once the clinical diagnosis was confirmed, IPK debridement was completed using a scalpel and number 15 blade, a podiatry drill and a spherical podiatry burr. In case of multiple IPKs, the patient chose the most painful lesion to be considered for the trial. IPK’s length and width was measured with a millimeter scale ruler used in wound care (**accuracy ± 0.1 mm)**. The depth was estimated with a cotton tip applicator technique measured with the same ruler [31]. If the patient was assigned to receive needle insertion or injection, a 27 gauge needle on a 3 mL syringe was inserted at 10 to 15 degrees with the bevel facing up approaching from the IPK’s right side. If the syringe’s piston was raised, the podiatrist pressed completely on it to inject 1 mL of the liquid without knowing its nature being either 0.9% sterile sodium chloride water or 2% (20 mg/ml) lidocaine solution (Aspen Pharma, Canada). IPK was then bandaged with a sterile gauze and medical tape (Hypafix, BSN medical). The patient was instructed to keep the dressing dry for 24 h before its removal. Patient were also advised to avoid any kind of treatment for their IPK between trial interventions. At each intervention, the podiatrist asked the patient if he or she received another treatment since the last visit. IPK photo was taken at each step of the intervention (before and after debridement and after the intervention). Photos of one patient are presented in Additional file 2 as an example. Each group was planned to receive four times the same intervention at a 4-week interval. A telephone follow-up was made at 6 months and 12 months after the first intervention.

### Data collection and outcomes measures

A questionnaire presented in Additional file 3 was performed at the first visit to collect baseline characteristics (e.g. demographic data, treatments already tried including frequency and effectiveness) by the podiatrist. These data were collected to compare our sample with the general population with IPKs. At each intervention (T2, T3 and T4), patients were asked by the research assistant to rate their pain level during the seven previous days on the visual analogue scale (VAS) [32]. The VAS is a 10 cm line anchored at the beginning by “no pain” and at the end by “worst pain imaginable”. The patient had to place a vertical mark on the scale to indicate his pain intensity level. VAS score is established by measuring the distance in centimeters (0 to 10) from the “no pain” anchor point [32]. Patients had to complete the validated foot pain questionnaire translated into French and shortened to 27 questions, the Foot-Function-Index-Revised (FFI-R) [33, 34]. The FFI-R is presented in Additional file 4. In order to make sure that the protocol was safe for patients according to CONSORT extension for harms and adverse events, the podiatrist ask if the patient had any adverse effects or complications secondary to last treatment [35]. The podiatrist was not allowed to ask patients about IPK’s symptoms and function during visits. A telephone follow-up was made 6 and 12 months after the first intervention (baseline) in order to complete the same questionnaire answered during the first visit (Additional file 2), VAS (pain level during the seven previous days) and FFI-R questionnaire (Additional file 4). Data collection at 6 and 12 months was completed with three additional questions about pain perceived during the experimental interventions as presented in Additional file 5.

### Statistical analysis

All non-parametric values (baseline characteristics) have been subjected to a chi-squared test. Pain intensity (VAS) and FFI-R scores were independently subjected to a repeated-measures analysis of variance (ANOVA x r) having four levels of group (scalpel debridement alone (group 1), scalpel debridement combined with needle insertion (group 2), scalpel debridement combined with subcutaneous injection of PSW (group 3) or scalpel debridement combined with subcutaneous injection of LS (group 4)) and five levels of time of measurements (baseline intervention, second intervention (T2), third intervention (T3), fourth intervention (T4), and 6-month (T6) and 12-month (T12) follow-ups). Statistical significance was set, for all analyses, at *p* value ≤0.05. All analyses were performed using Statistica (version 13; StatSoft; Oklahoma, United States) and Excel (version 16; Microsoft Corporation; Washington, United Stated). Finally, we did an intention-to-treat analysis since missing data by dropped-out patients were replaced by the last value available. Feasibility data are reported with descriptive statistics in a narrative synthesis. Finally, this feasibility study is reported using CONSORT extension for pilot and feasibility trials checklist [36]. The study protocol was retrospectively registered on Open Science Framework (osf.io/srqp9) and in ClinicalTrial.gov (NCT 04777227).

## Results

### Feasibility

As showed in Fig. 1, 40 patients were recruited over a two-year period in the two clinical settings. Recruitment was stopped because four equal randomized groups of 10 patients were enrolled. The same podiatrist (MPM) provided all 154 interventions and placed all follow-up phone calls at T6 and T12. Ninety percent of patients (*n* = 36) attended all four prescribed interventions and completed the 6-month follow-up. However, only 67.5% (*n* = 27) completed the 12-month follow-up. Several attempts had been made by the podiatrist to reach patients by phone, but efforts remained unfruitful to collect follow-up data in some cases. Throughout the protocol, the major reason for dropout was anticipated pain that would be provoked by the injection. At the 6-month follow-up phone call, 12.5% of group 2, 30% of group 3 and 14.3% of groups 4 patients said that the effectiveness of the treatments was not worth the pain felt during the interventions. The pain was felt during the needle insertion or the injection. Furthermore, some patients still had discomfort few days after the intervention. No patient in the group 1 reported pain secondary to the intervention (debridement only). Except for pain, no patient reported any other side effects for the duration of the feasibility study related to the intervention.

### Safety

Only pain at the injection site for a few hours have been stated by a limited number of patients. No other adverse effect has happened.

### Group comparisons

Patient baseline characteristics are presented in the Table 2. There was no statistically significant difference between groups (*p* > 0.05). They were equivalent for age, time since apparition of IPK, time before first consultation, time spent standing up per day and daily cigarette smoking. Women represented 57.5% (23/40) of all patients. IPKs selected for study intervention were located under a metatarsal head, the styloid process of the fifth metatarsal and the heel respectively for 87.5% (34/40), 7.5% (3/40) and 5% (2/40). Thirty-five percent (14/40) of patients wore plantar orthoses on a daily basis and 37.5% (15/40) had at least one foot deformity. It was observed by the podiatrist during the first evaluation (baseline) that 11 of the 14 smokers (78.5%) and 4 of the 22 non-smokers (18%) presented macerated skin around the selected. Based on patient self-reporting data, none received another intervention than what was provided during the trial.

**Table 2:** Patients’ baseline characteristics

Number of patients (Total = 40)	Group 1 *n* = 10	***Group 2*** *n* = 10	Group 3 *n* = 10	Group 4 *n* = 10	***P***^**a**^ value
Age in years, Mean (SD)	59.7 (18.8)	60.1 (14.8)	61.6 (14.4)	50.3 (17.7)	0.4
Sex ratio, M:W	6:4	3:7	4:6	4:6	–
Time since IPK apparition in months, Mean (SD)	213.0 (228.3)	192.6 (140.9)	132.0 (74.7)	62.8 (69.2)	0.1
Time before first consultation in weeks, Mean (SD)	439.4 (602.4)	153.4 (198.6)	242.0 (503.5)	119.2 (201.2)	0.3
Time spent standing hours/day, Mean (SD)	6.5 (2.9)	6.0 (2.3)	7.9 (3.2)	6.9 (3.2)	0.5
Debridement frequency before study in days, Mean (SD)	100.9 (102.2)	142.8 (134.4)	98.9 (29.0)	91.3 (38.3)	0.6
Smoking (n)	6	2	2	4	0.2
Plantar orthoses worn daily (n)	5	3	4	2	0.5
Macerated IPK at first visit (n)	6	3	3	3	0.4
Presence of foot deformity (n)	3	6	4	2	0.3
IPK location ratio, Met:H:S	10:0:0	10:0:0	7:2:1	7:0:2	–

^a^ *P*-value calculated with the chi-squared test

*SD* standard deviation; *M* men; *W* women; *IPK* intractable plantar keratoma; *Met* under metatarsal head; *H* under the heal; *S* under styloid process of the 5th metatarsal; *n* number

### Clinical outcomes

**Fig. 2:**
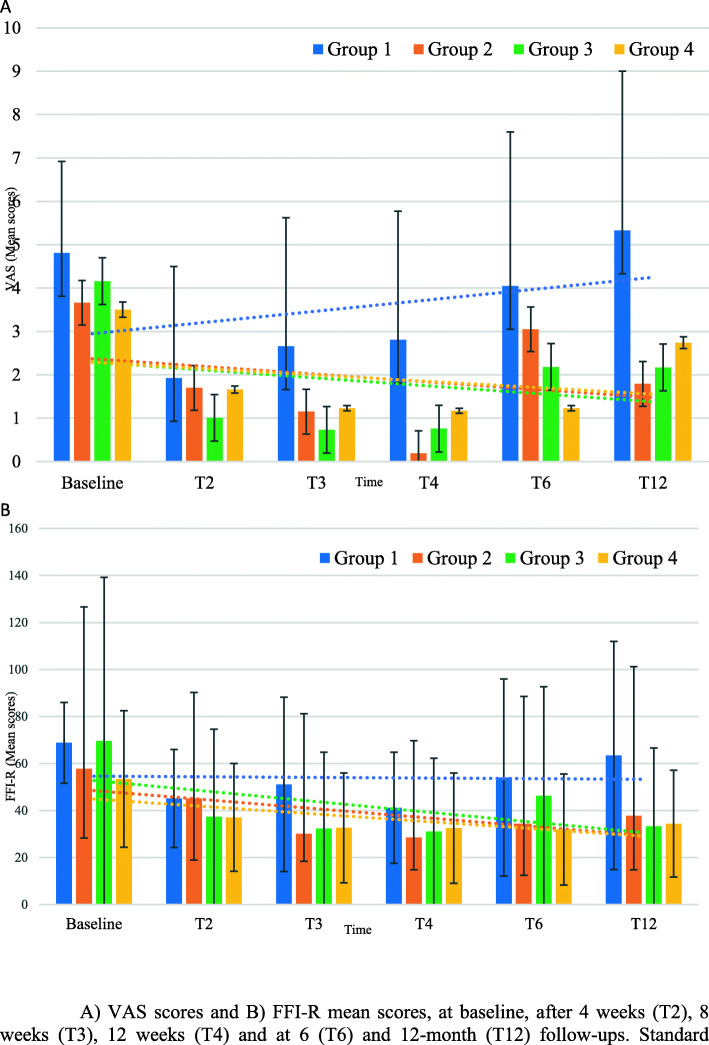
**a** VAS scores and **b** FFI-R mean scores, at baseline, after 4 weeks (T2), 8 weeks (T3), 12 weeks (T4) and at 6 (T6) and 12-month (T12) follow-ups. Standard deviations are represented in box plots. Trendlines associated with each group are represented as dashed lines

Clinical outcomes data are detailed in Table 3. After analysis, there were no statistically significant difference between groups for any clinical outcomes: VAS pain score (F [3, 36] = 1.45, *p* = 0.26), FFI-R score (F [3, 36] = 0.85, *p* = 0.33), IPK’s width (F(3,36) = 0.55, *p* = 0.65), IPK’s length (F [3, 36] = 0.36, *p* = 0.78) and IPK’s depth (F [3, 36] = 1.25, *p* = 0.31). However, analysis revealed an effect of time on VAS (F [4, 48] = 18.03, *p* < 0.001), FFI-R score (F [4, 48] = 17.85, *p* < 0.001), IPK’s width (F [3, 36] = 12.74, *p* < 0.001), IPK’s length (F [3, 36] = 5.93, *p* = 0.001) and IPK’s depth (F [3, 36] = 51.94, *p* < 0.001). Trends for outcomes measures VAS and FFI-R are presented in Fig. 2. Similarly, no statistically significant Group x Time interaction was found on VAS (F(12,144) = 1.12, *p* = 0.83), FFI-R score (F(12,144) = 0.93, *p* = 0.35), IPK’s width (F(9,108) = 1.40, *p* = 0.20), IPK’s length (F(9,108) = 1.27, *p* = 0.26) and IPK’s depth (F(9,108) = 1.41, *p* = 0.19).

**Table 3:** Clinical outcomes data by group

		Group 1		Group 2		Group 3		Group 4	
		Mean (SD)		Mean (SD)		Mean (SD)		Mean (SD)	
		*n* = 10 at baseline		*n* = 10 at baseline		*n* = 10 at baseline		*n* = 10 at baseline	
VAS	Baseline	4.8	(2.1)	3.7	(2.0)	4.2	(2.9)	3.5	(3.0)
(0–10)	T2	1.9	(2.6)	1.7	(2.3)	1.0	(1.6)	1.7	(2.3)
	T3	2.6	(3.0)	1.2	(2.1)	0.7	(1.3)	1.2	(2.3)
	T4	2.8	(3.0)	0.2	(0.3)	0.6	(1.4)	1.2	(2.3)
	T6	4.0	(3.6)	3.1	(3.0)	2.2	(3.1)	1.2	(2.3)
	T12	5.3	(3.7)	1.8	(2.7)	2.2	(2.4)	2.7	(2.8)
FFI-R	Baseline	68.8	(17.1)	57.8	(29.4)	69.6	(34.7)	53.4	(29.6)
(/270)	T2	45.1	(20.9)	45.1	(26.2)	37.3	(25.8)	37.1	(22.9)
	T3	51.1	(37.1)	30.1	(11.7)	32.4	(22.6)	32.6	(23.4)
	T4	41.2	(23.7)	28.5	(13.7)	31.1	(24.8)	32.5	(23.4)
	T6	54.1	(41.9)	34.4	(21.9)	46.3	(42.4)	31.9	(23.6)
	T12	63.5	(48.5)	37.7	(22.9)	33.3	(14.0)	34.4	(22.7)
IPK’s Width	Baseline	3.4	(1.6)	3.3	(2.7)	3.1	(1.6)	2.0	(1.5)
(mm)	T2	2.8	(0.9)	2.1	(1.3)	2.8	(2.6)	2.1	(0.9)
	T3	2.1	(0.9)	1.7	(1.5)	2.5	(3.4)	1.4	(1.4)
	T4	2.1	(1.4)	1.9	(1.6)	0.9	(1.1)	1.4	(1.4)
IPK’s Length	Baseline	2.7	(1.0)	2.9	(2.7)	3.4	(2.2)	2.5	(1.5)
(mm)	T2	2.7	(1.0)	2.1	(1.6)	2.8	(2.6)	2.1	(1.2)
	T3	2.5	(1.1)	1.9	(1.4)	3.0	(4.1)	1.6	(1.6)
	T4	2.3	(1.5)	1.8	(1.4)	1.1	(1.5)	1.6	(1.6)
IPK’s Depth	Baseline	1.7	(0.7)	1.7	(0.8)	1.7	(0.6)	1.5	(0.9)
(mm)	T2	1.4	(0.5)	1.1	(0.9)	0.8	(0.4)	1.0	(0.7)
	T3	0.9	(0.1)	0.8	(0.7)	0.3	(0.3)	0.7	(0.8)
	T4	1.0	(0.7)	0.8	(0.6)	0.3	(0.4)	0.7	(0.8)

*VAS* Visual analog scale; *FFI-R* Foot Function Index-Revised; *IPK* Intractable Plantar Keratoma; *SD* Standard Deviation; mm: millimeter; *T2* intervention at 4-week; *T3* intervention at 8-week; *T4* intervention at 12-week; *T6* 6-month follow-up; *T12* 12-month follow-up

## Discussion

The objectives of this study were to determine feasibility, safety and gather preliminary outcomes in relation with four IPK treatment combinations, including scalpel debridement alone, scalpel debridement combined with needle insertion, scalpel debridement combined with subcutaneous injection of PSW or scalpel debridement combined with subcutaneous injection of LS. This study was conducted to explore new treatments for IPK. Regarding feasibility, the main comment from groups 2, 3 and 4 patients was that pain felt during the intervention was important and that it was not worth treatment’s overall efficacy. This was stated by more that 55% of the patients respectively at 6 and 12-month follow-ups. To avoid this situation, an anesthetic block of the tibialis nerve could have been performed before the needle insertion and injection, but callus treatment does not represent a current indication for this procedure [37]. Furthermore, the fact that twice the proportion of patients in group 3, whom received a saline water injection, had more pain than patients in group 4, whom received a LS injection, might possibly be explained by the anesthetic property of lidocaine [38]. This should be investigated in a future study.

The treatment combinations have been determined by a literature review, previous work and hypotheses [20, 39]. Many aspects of those combinations could explain the time effect observed in this study. In ascending order, intradermic needle insertion, PSW and LS injection all increase local blood flow [40, 41]. Furthermore, microneedling, which consists in needle insertion in scar or tendon, has been shown effective in diminishing acne scar and in treatment of Achilles tendinitis and lateral epicondyloses [42, 43]. This technique creates micro-wounds, restarts normal healing process and stimulates collagen and elastin production, which are important components of skin structure. Furthermore, lidocaine has a negative effect on fibroblast proliferation in cell culture medium [44, 45]. The fibroblasts are dermal cells that continually interact with keratinocytes through growth factors and dictate their proliferation rate [46]. These effects have been observed at clinically-used concentrations [44, 47]. Finally, lidocaine modifies granulocytes cellular membrane [48, 49]. Since the membranes are modified, these cells can no longer adhere to surfaces, which prevents them from releasing inflammatory factors [49]. This interesting property of lidocaine could therefore possibly reduce the pain associated with IPKs. However, those explanations are hypotheses, and histopathological and biochemistry analyses could have been relevant but are challenging and not a part of an IPK clinical treatment goals.

The VAS and the IPK measurements taken at each follow-up interventions furthermore show a downward trend in patients who received an injection (either group 3 or 4) compared with the other groups. The FFI-R score demonstrated a higher tendency to produce low scores with the PSW and LS injection interventions. Also, patients who had scalpel debridement only reported higher mean of VAS, FFI-R score and IPK measurements. Those results are not statistically significant, but this can possibly be explained by the small number of patients in each group. It is also important to note the shorter interval of time between treatments planned during the experimental protocol in comparison with what the patients where used to before this study. In fact, during the study all patients had a scalpel debridement every 28 to 30 days, compared with reported previous debridement frequency at an average interval of 108.5 days all groups combined. The statistically significant time effect could be explained by the fact that all treatments were highly effective after the first intervention and as a result of higher treatment frequency. The shorter period of time between treatments leads to a decrease in hyperkeratosis’ accumulation. Since the accumulation is less, this could explain the lower scores obtained on VAS and FFI-R. These results cannot be directly compared with other studies on the subject as this project is the first one comparing all treatment combinations with scalpel debridement alone for IPKs. However, pain alleviation after callus debridement including IPK, is reported for short and medium terms and are consistent with our results [15, 23, 50].

### Strengths and limitations

The strength of this study is its innovative quality as it explores potential IPKs treatments that could be long-lasting and its contribution to raise new hypotheses for people who suffer from this prevalent foot health problem [51]. Regarding efficacy, the limited number of patients included limits results interpretation. However, this feasibility study provided information about recruitment, retention, randomization and treatment fidelity [51]. The recruitment was not as easy and fast as anticipated. This can be explained by the nature of the experimental treatment and this knowledge could help planning for future studies on IPK treatment with injection. For some patients, the use of a needle can be a major barrier to treatment and seems too invasive for how they perceive their condition [52]. Even though the recruitment was conducted on a two-year period, only 40 patients were recruited. Initially, the ideal target was 60 (15 patients per groups). Another reason justifying recruitment difficulties is the fact that for some individuals, the initial foot pain and limitations were low so the pain anticipated from the needle insertion and injection may not be worth the potential pain relief compared to debridement alone. In addition, the pain felt during intervention with needle insertion played a role in patient retention. The randomization was feasible, but it was not possible to blind (patient and podiatrist) of the group 1 (debridement only) comparing to the three other groups. In this study, the evaluator and the performer were the same person which can lead to performance bias. However, to limit this risk of bias, the treatment containers were always covered during the evaluation before the experimental treatment was provided to the patient. The syringes content was also masked by an assistant. Even if 4 weeks separated each treatment, it would have been possible to remember if the patient received scalpel debridement only or another treatment because of the small sample size. Finally, the ruler that measured IPK’s length, width and depth could lead to an increased measurement uncertainty and lack of repeatability. The use of a digital caliper could have help but the depth measurement would have still been a challenge. In a further study, a more efficient tool is needed to analyse continuous variable like IPK’s size or this outcome could be addressed as a dichotomous variable like IPK cured or not. Overall, there are remaining uncertainties about the feasibility to use PWS and LS injections for IPK treatment.

In conclusion, the treatment by needle insertion, PSW injection or LS injection of painful IPKs is feasible and safe. Findings has demonstrated a trend towards diminished pain intensity on VAS and FFI-R compared to scalpel debridement alone. On the other hand, despite this observed trend, there was no statistically significant difference in the final scores (VAS and FFI-R) between all groups. Further research with strongest design including more patients need to be done before considering the use of an injection therapy for painful IPKs.

## Supplementary Information

**Additional file 1:** *Patient selection questionnaire (french version). (DOCX 20 kb)*

**Additional file 2:** *Patient #30 photos: Example of an IPK receiving an injection. (DOCX 182 kb)*

**Additional file 3:** *Patient Interview (french version). (DOCX 21 kb)*

**Additional file 4:** *Foot-Function-Index-Revised (French version). (DOCX 24 kb)*

**Additional file 5:** *Questions about the pain perceived during the experimental interventions at 6 and 12-month follow-ups (French version). (DOCX 21 kb)*

## Data Availability

Raw data will be available on request.

## Abbreviations

IPK: Intractable Plantar Keratoma
VAS: Visual Analog Scale
FFI-R: Foot Function Index Revised
H: Heel
HA: Hyaluronic acid
HAV: Hallux-Abducto-Valgus
HT: Hammer toe
M: Men
Met: Metatarsal
N: Number
PSW: Physiological saline water
LS: Lidocaine solution
S: Styloid process
SD: Standard deviation
W: Women
