# Supplementary material for: Effectiveness of saline water and lidocaine injection treatment of intractable plantar keratoma: a randomised feasibility study
Source: J Foot Ankle Res. 2021 Apr 13;14:30. doi: 10.1186/s13047-021-00467-7 (PMC8042939; doi:10.1186/s13047-021-00467-7)
Supplement: Supplementary file 1 — Additional file 1: Patient selection questionnaire (french version). (DOCX 20 kb) [file 13047_2021_467_MOESM1_ESM.docx]

*Patient selection questionnaire (french version)*

**Patient identification : ____________________________**

|  | OUI | NON |
| --- | --- | --- |
| Souffrez-vous de la présence de cors plantaires (cors, durillons) à la plante de vos pieds depuis 3 mois ou plus? |  |  |
| Avez-vous 18 ans ou plus? |  |  |
| Pourriez-vous être enceinte ou êtes-vous allaitante? |  |  |
| Souffrez-vous de troubles vasculaires périphériques? |  |  |
| Souffrez-vous de troubles neurologiques (incluant les neuropathies périphériques associées au diabète)? |  |  |
| Prenez-vous une médication pour éclaircir votre sang, soit une médication antiplaquettaire (p. ex. Aspirine, Asaphen) ou anticoagulante (p. ex. Coumadin) de façon régulière? |  |  |
| Prenez-vous une médication (p. ex. Cyclosporine, Méthotrexate) ou souffrez-vous d’une condition médicale (p. ex. cancer, VIH) pouvant affecter votre système immunitaire? |  |  |
| Avez-vous une allergie connue aux anesthésiants locaux ? |  |  |
| Lorsque vous vous blessez, est-ce que la plaie cicatrise normalement? |  |  |
| Présentez-vous actuellement une plaie, un ulcère ou une infection à l’un de vos pieds? |  |  |
| Souffrez-vous présentement de douleurs aux pieds autres que celles associées à la présence de cor plantaire (p. ex. fasciite/syndrome de l’épine de Lenoir, névrome de Morton, tendinite, etc.)? |  |  |
