# Supplementary material for: Effectiveness of saline water and lidocaine injection treatment of intractable plantar keratoma: a randomised feasibility study
Source: J Foot Ankle Res. 2021 Apr 13;14:30. doi: 10.1186/s13047-021-00467-7 (PMC8042939; doi:10.1186/s13047-021-00467-7)
Supplement: Supplementary file 2 — Additional file 2: Patient #30 photos: Example of an IPK receiving an injection. (DOCX 182 kb) [file 13047_2021_467_MOESM2_ESM.docx]

*Patient #30 photos: Example of an IPK receiving an injection*
