# Supplementary material for: Effectiveness of saline water and lidocaine injection treatment of intractable plantar keratoma: a randomised feasibility study
Source: J Foot Ankle Res. 2021 Apr 13;14:30. doi: 10.1186/s13047-021-00467-7 (PMC8042939; doi:10.1186/s13047-021-00467-7)
Supplement: Supplementary file 3 — Additional file 3: Patient Interview (french version). (DOCX 21 kb) [file 13047_2021_467_MOESM3_ESM.docx]

*Patient Interview (french version)*

**ENTREVUE INDIVIDUELLE**

**Patient identification: ______________________________________________
Date :_________________________________________**

Moment d’apparition des callosités :___________________________________________

Contexte d’apparition connu (idiopathique, cicatrice traumatique/iatrogène, verrue, chaussure, etc.) : __________________________________________________________

Chronologie (graduel/soudain, ordre d’apparition si lésions multiples) :________________

Délai avant le diagnostic :____________________________________________________

Qui a référé en podiatrie :___________________________________________________

Histoire familiale de troubles similaires :_______________________________________

________________________________________________________________________

Traitement actuel :________________________________________________________

_______________________________________________________________________

Estimation du coût du traitement actuel (monétaire et temps) :______________________

Autres traitement essayé et résultat obtenu :

- Coussinage :_______________________________________________________
- Pierre ponce :______________________________________________________
- Crème hydratante/émoliente :_________________________________________
- Orthèses plantaires :_________________________________________________
- Acide pyrogallique :_________________________________________________
- Kératolytiques :_____________________________________________________
- Chirurgie tissus mous :_______________________________________________
- Chirurgie osseuses :_________________________________________________
- Injections :_________________________________________________________
- Débridement maison :________________________________________________
- Autres :___________________________________________________________

Histoire d’ulcère :_________________________________________________________

Estimation du nombre d’heures debout par jour :_________________________________

Type de chaussures (travail, intérieur, extérieur) :________________________________

Activités pratiquées et nombre d’heures pour chacune :___________________________

Maladies connues :________________________________________________________

________________________________________________________________________________________________________________________________________________

Traitements de ces maladies le cas échéant (interventions, médication, etc.) :

________________________________________________________________________________________________________________________________________________________________________________________________________________________

Médication régulière :______________________________________________________

________________________________________________________________________________________________________________________________________________

Produits naturels/suppléments/vitamines :______________________________________

Allergies connues (médication, environnement, alimentaires) :______________________

Fumeur :________________________________________________________________

Consommation d’alcool :___________________________________________________

Consommation de café/thé :_________________________________________________

Intérêt pour un traitement par injection 4 fois/1-2 ans à la place du débridement fréquent :________________________________________________________________

Droitier ou gaucher :_______________________________________________________

Chute au cours des 2 dernières années :________________________________________

Poids et taille :____________________________________________________________
