# Supplementary material for: Effectiveness of saline water and lidocaine injection treatment of intractable plantar keratoma: a randomised feasibility study
Source: J Foot Ankle Res. 2021 Apr 13;14:30. doi: 10.1186/s13047-021-00467-7 (PMC8042939; doi:10.1186/s13047-021-00467-7)
Supplement: Supplementary file 4 — Additional file 4: Foot-Function-Index-Revised (French version). (DOCX 24 kb) [file 13047_2021_467_MOESM4_ESM.docx]

*Foot-Function-Index-Revised (French version)*

Patient identification : _________________________

**Pour chacun des énoncés dans les 4 sections, veuillez encercler ou cocher la case correspondant à vos impressions concernant la semaine précédente.**

1. **DOULEUR AUX PIEDS**

AU COURS DE LA DERNIÈRE SEMAINE, comment qualifiez-vous la sévérité de vos douleurs de pieds ?

|  |  | Aucune douleur |  |  |  |  |  |  |  |  | Pire douleur imaginable |
| --- | --- | --- | --- | --- | --- | --- | --- | --- | --- | --- | --- |
| 1 | La pire douleur ressentie aux pieds ? | 1 | 2 | 3 | 4 | 5 | 6 | 7 | 8 | 9 | 10 |
| 2 | La douleur aux pieds le matin ? | 1 | 2 | 3 | 4 | 5 | 6 | 7 | 8 | 9 | 10 |
| 3 | La douleur aux pieds à la marche pieds nus ? | 1 | 2 | 3 | 4 | 5 | 6 | 7 | 8 | 9 | 10 |
| 4 | La douleur aux pieds en position debout pieds nus ? | 1 | 2 | 3 | 4 | 5 | 6 | 7 | 8 | 9 | 10 |
| 5 | La douleur aux pieds à la marche avec souliers ? | 1 | 2 | 3 | 4 | 5 | 6 | 7 | 8 | 9 | 10 |
| 6 | La douleur aux pieds en position debout avec souliers ? | 1 | 2 | 3 | 4 | 5 | 6 | 7 | 8 | 9 | 10 |
| 7 | La douleur aux pieds à la marche avec vos orthèses ? | 1 | 2 | 3 | 4 | 5 | 6 | 7 | 8 | 9 | 10 |
| 8 | La douleur aux pieds en position debout avec vos orthèses ? | 1 | 2 | 3 | 4 | 5 | 6 | 7 | 8 | 9 | 10 |
| 9 | La douleur aux pieds à la fin d’une journée habituelle ? | 1 | 2 | 3 | 4 | 5 | 6 | 7 | 8 | 9 | 10 |

1. **RÉALISATION DE TÂCHES QUOTIDIENNES**

AU COURS DE LA DERNIÈRE SEMAINE, comment qualifiez-vous la difficulté à réaliser certaines tâches ?

|  |  | Aucune difficulté |  |  |  |  |  |  |  |  | Trop difficile/Irréalisable |
| --- | --- | --- | --- | --- | --- | --- | --- | --- | --- | --- | --- |
| 10 | Difficulté à vous déplacer dans la maison ? | 1 | 2 | 3 | 4 | 5 | 6 | 7 | 8 | 9 | 10 |
| 11 | Difficulté à marcher à l’extérieur de la maison ? | 1 | 2 | 3 | 4 | 5 | 6 | 7 | 8 | 9 | 10 |
| 12 | Difficulté à marcher quatre coins de rue ? | 1 | 2 | 3 | 4 | 5 | 6 | 7 | 8 | 9 | 10 |
| 13 | Difficulté à monter les escaliers ? | 1 | 2 | 3 | 4 | 5 | 6 | 7 | 8 | 9 | 10 |
| 14 | Difficulté à descendre les escaliers ? | 1 | 2 | 3 | 4 | 5 | 6 | 7 | 8 | 9 | 10 |
| 15 | Difficulté à vous tenir sur la pointe des pieds ? | 1 | 2 | 3 | 4 | 5 | 6 | 7 | 8 | 9 | 10 |
| 16 | Difficulté à vous lever d’une chaise ? | 1 | 2 | 3 | 4 | 5 | 6 | 7 | 8 | 9 | 10 |
| 17 | Difficulté à monter sur le trottoir ? | 1 | 2 | 3 | 4 | 5 | 6 | 7 | 8 | 9 | 10 |
| 18 | Difficulté à marcher rapidement ? | 1 | 2 | 3 | 4 | 5 | 6 | 7 | 8 | 9 | 10 |

1. **LIMITATION DANS LES ACTIVITÉS**

AU COURS DE LA DERNIÈRE SEMAINE, comment qualifiez-vous ces aspects de votre journée ?

|  |  | Jamais |  |  |  |  |  |  |  |  | Tout le temps |
| --- | --- | --- | --- | --- | --- | --- | --- | --- | --- | --- | --- |
| 19 | Le temps passé à l’intérieur à cause de votre problème de pieds ? | 1 | 2 | 3 | 4 | 5 | 6 | 7 | 8 | 9 | 10 |
| 20 | Le temps passé au lit à cause de votre problème de pieds ? | 1 | 2 | 3 | 4 | 5 | 6 | 7 | 8 | 9 | 10 |
| 21 | Limiter vos activités à cause de votre problème de pieds ? | 1 | 2 | 3 | 4 | 5 | 6 | 7 | 8 | 9 | 10 |
| 22 | Utiliser une canne, des béquilles ou une marchette à l’intérieur ? | 1 | 2 | 3 | 4 | 5 | 6 | 7 | 8 | 9 | 10 |
| 23 | Utiliser une canne, des béquilles ou une marchette à l’extérieur ? | 1 | 2 | 3 | 4 | 5 | 6 | 7 | 8 | 9 | 10 |
