# Supplementary material for: Effectiveness of saline water and lidocaine injection treatment of intractable plantar keratoma: a randomised feasibility study
Source: J Foot Ankle Res. 2021 Apr 13;14:30. doi: 10.1186/s13047-021-00467-7 (PMC8042939; doi:10.1186/s13047-021-00467-7)
Supplement: Supplementary file 5 — Additional file 5: Questions about the pain perceived during the experimental interventions at 6 and 12-month follow-ups (French version). (DOCX 21 kb) [file 13047_2021_467_MOESM5_ESM.docx]

*Questions about the pain perceived during the experimental interventions at 6 and 12-month follow-ups (French version)*

Patient identification : _________________________

Date :______________________________________

QUESTIONS À RÉPONDRE POST-SIX MOIS / POST 12 MOIS.

**J’ai 2 questions à vous poser concernant votre expérience face au traitement. Vous devez répondre en utilisant l’échelle suivante :**

1. Tout à fait d’accord
2. D’accord
3. Ni en désaccord ni d’accord
4. Pas d’accord
5. Pas du tout d’accord
6. Considérez-vous que l’effet du traitement reçu vaut la douleur ressentie lors de celui-ci? Rép : ___
7. Si le participant était dans le groupe 1 : Présentement, dans son ensemble, considérez-vous le traitement reçu lors de la recherche comme efficace? Rép : ___
8. Si le participant était dans le groupe 2-3 ou 4 : Présentement, dans son ensemble, considérez-vous le traitement par injection comme efficace? Rép : ___

**À répondre par oui ou par non :**

1. Avez-vous eu recours à des soins pour le cor plantaire depuis le dernier rendez-vous? Rép : ___
2. Avez-vous eu besoin de faire des débridements maison depuis le dernier rendez-vous? Rép : ___

**Sur une échelle de 0 à 10, 0 étant aucune douleur, 10 étant la pire douleur imaginable, pouvez-vous grader votre douleur ressentie au cor plantaire dans les 7 derniers jours?** Rép : ___
